# Developmental Eye Movement (DEM) Test in Adults: Age-Related Changes and Italian Normative Data

**DOI:** 10.3390/vision9010010

**Published:** 2025-02-02

**Authors:** Alessio Facchin, Silvio Maffioletti, Roberta Daini

**Affiliations:** 1 Neuroscience Research Center, Department of Medical and Surgical Sciences, Magna Graecia University, 88100 Catanzaro, Italy; 2 Department of Psychology, University of Milano-Bicocca, 20126 Milan, Italy; roberta.daini@unimib.it; 3 Institute of Research and Studies in Optics and Optometry, 50059 Vinci, Italy; silvio.maffioletti@unito.it; 4 Degree Course of Optics and Optometry, University of Torino, 10124 Torino, Italy; 5 COMiB—Optics and Optometry Research Center, University of Milano-Bicocca, 20126 Milan, Italy; 6 IRCCS Fondazione Don Carlo Gnocchi ONLUS, 20126 Milan, Italy

**Keywords:** DEM test, concussion, norms, neuropsychological test, oculomotor dysfunction

## Abstract

The developmental eye movement (DEM) test is a paper-based tool used to assess ocular motor skills in children. By naming numbers in a simple and easy simulated reading task, the DEM test provides an oculomotor efficiency score without complex eye-tracking equipment. Studies have shown that its usefulness can be extended to adults, despite its name suggesting that it is used primarily for developmental ages. However, for a broader application in the adult population in a clinical setting, there are no adult-specific norms. This study aimed to develop adult normative data for the Italian-speaking population and assess whether the DEM scores were influenced by age. In this study, 521 healthy Italian adults, aged 20 to 79 years, participated. Normative data were obtained by using a regression-based framework with demographic variables as predictors. Results show that age influences all sub-measures of time such as Vertical Time (VT), Adjusted Horizontal Time (AHT), and the Ratio score, but did not influence the error score. This is in line with the processing speed reduction in aging. Errors were influenced by education and gender. DEM norms, defined and scored using percentiles and equivalent scores, allow the assessment of oculomotor efficiency in adults, making this test suitable for use in all clinical settings, particularly in neuropsychological and neurological ones.

## 1. Introduction

Eye movement disorders, also referred to as oculomotor dysfunction, oculomotor anomalies, or oculomotor deficits, are common manifestations of various neurological disorders [1,2].

They range from 11% to 95% of patients, depending on the underlying disease [3,4,5]. According to a study by Thiagarajan et al. [6], which included more than 200 patients, 90% of those with traumatic brain injury and 86% of those with cerebrovascular accidents had some form of oculomotor dysfunction. Also, many ocular movement disorders can be observed in cerebellar patients [7].

One of the first clinical manifestations of inherited and acquired neurodegenerative diseases is eye movement disorders, which play a crucial role in their diagnosis. The degenerative processes in neurodegenerative disorders including Parkinson’s disease, Huntington’s disease, and multiple sclerosis frequently affect the brain circuits that control eye movements, resulting in abnormalities in oculomotor function [8,9].

Oculomotor deficits provide valuable information about the pathophysiology of these conditions, aiding in diagnosis and providing potential therapeutic targets [3,10]. Consequently, the clinical assessment of oculomotor function can help to differentiate diagnoses [11,12], while electrophysiological measures provide useful biomarkers for understanding disease pathophysiology and progression.

The most accurate instrument used to assess eye movement disorders is the eye tracker [13]; however, larger anomalies can be detected clinically by the direct observation of eye movements [14].

However, not all clinicians have the possibility to use an eye tracker for diagnosis, and the interpretation of the results is not easy or fast. As such, a series of other indirect evaluations of eye movement modalities have been developed over the years [15]. Two paper-based psychometric instruments are of particular interest. Both are based on a series of cards in which the patient is asked to name numbers arranged in different ways, including one structured in a reading-like condition.

The King–Devick (KD) test is a rapid visual screening tool in which an individual reads numbers aloud quickly from test cards or using a computer-based application. It permits the indirect assessment of underlying brain trauma such as concussion via impairments in saccadic rhythm [16]. Specifically, its application has gained relevance in the context of sports traumatic brain injury and concussion [17,18].

The developmental eye movement (DEM) test is a paper-based oculomotor test designed to identify oculomotor dysfunction in children by using a similar procedure used in the KD [15,19].

The task requires naming numbers in a reading-like condition. The DEM test comprises a pretest card and three test cards, where numbers are organized in vertical columns or in horizontal rows like a reading text.

The Vertical Time (VT) is the sum of the time of the two cards A and B. The VT reflects the time taken to read aloud 80 numbers. The Adjusted Horizontal Time (AHT) is the time of card C adjusted for the omission or addition of errors. AHT represents the time required to read aloud a horizontal pattern of 80 numbers as well as the time required to perform saccadic eye movements. The AHT is divided by the VT to calculate the Ratio score. The error score reflects the total number of errors made on the C card.

The test also has a relatively straightforward interpretation. A Ratio value is used to evaluate ocular motility dysfunctions, while a VT value is used to diagnose naming problems. As reported in the manual [20], the combinations of the three DEM scores could lead to four different profiles: a normal condition (normal score on VT, AHT, and Ratio), a naming problem (low scores on VT and AHT and normal Ratio), an oculomotor problem (normal score on VT, but lower on AHT and Ratio) and a combined naming and oculomotor problem (lower scores on VT, AHT, and Ratio). However, the clinical diagnosis based on the dichotomization of each subtest result (positive/negative), and the discussion of which cutoff should be used (15th vs. 30th percentile) [21], has moved to a more dynamic interpretation of the results based on percentile scores [15].

Compared to KD, the DEM test has the advantage of considering the patient’s naming abilities during the evaluation process, which makes DEM a superior test [22,23]. This aspect enables it to differentiate between naming-related problems from oculomotor-related problems [15,19].

Since the DEM and KD tests measure similar constructs in healthy individuals, it follows that the DEM test can also be used in concussed patients and adults [24].

Thanks to its nature (and name), the DEM test has gained success in the evaluation of learning-related visual problems [15,25]. Originally developed for use in children with learning disabilities, this test can also be useful in identifying saccadic dysfunction in adults in general [23], and in adults with acquired brain injury. This tool is an inexpensive and easy method of assessing a particular aspect of the oculomotor function [26]. Even though the term “developmental” may be confusing, its use cannot be confined to the pediatric population.

Since there is a high frequency of oculomotor disturbances in neurological patients [27], the DEM test could be useful for clinicians that have no access to sophisticated eye trackers or need a fast assessment. In fact, the DEM test has already been used in mild traumatic brain injury patients for the assessment of oculomotor rehabilitation [28], in patients with multiple sclerosis with heterogeneous oculomotor disturbances [22] and in a group of various neurologic disorders [29], confirming its usefulness both in diagnosis and rehabilitation settings [30].

Although some studies have criticized DEM scores for their lack of strict correlation with specific eye movement parameters [31,32,33], the DEM does reflect clinically relevant factors. For instance, DEM performance relates to the level of academic performance [19,34,35,36], reading rate [37,38,39,40], and speed of visual processing [31,33], and permits the correct differentiation of oculomotor problems in developmental age [32,39]. Its psychometric properties were evaluated in the original publication, and successive studies showed only some limitations in short-term repeatability [41].

From different studies and reliable clinical applications, the need for adult norms for the DEM test has emerged [15,26,42,43]. Moreover, there are some pieces of evidence that DEM scores change with age [44,45]. Reading abilities seem to decline with age [46,47], so one expectation is to find an increased naming time in the older population.

The clinical implications of specific adult norms for the DEM test lie in the possibility of being able to detect an oculomotor disorder directly during neurological or neuropsychological evaluation without requiring complex eye-tracking equipment.

For broader use in the adult population such as neurological applications, specific norms for adults are lacking, while for developmental age, norms are available in 10 different languages, including Italian [48]. This study aims to assess age-related changes and develop adult norms for the DEM test using the well-established procedure of regression-based norm construction in neuropsychological tests.

## 2. Materials and Methods

### 2.1. Participants

The sample size required for this study was determined by a power analysis. Based on a regression model with three independent demographic variables (age, education, and sex), using α = 0.05, power = 0.80, and a small effect size f^2^ = 0.03, a minimum sample of 368 participants was required.

Inclusion and exclusion criteria: Participants were native Italians and Italian speakers without any history of current or past neurological or psychiatric disorders (including stroke, brain injury, clinically diagnosed dementia, depression, alcohol or drug abuse), and current eye disease. In order to exclude dementia or mild cognitive impairment, a normal score on the Mini-Mental State Examination (MMSE; adjusted score for demographic variables > 23.8, Italian norms [49]) was required. A corrected near visual acuity equal to or better than +0.2 LogMAR (0.63 decimal) using a Goodlite 729000 card (https://good-lite.com/ (accessed on 1 February 2025)) was requested.

A convenience sample of volunteers was selected from direct contacts of the examiners (from the Lombardy, Piedmont, Liguria, Veneto, Tuscany, and Campania regions of Italy). No compensation was given. Initially, a sample of 525 participants was collected, but 4 participants did not meet the inclusion criteria (2 for lower MMSE score and 2 for lower visual acuity). A balance was attempted in the collection of data (Table 1), considering the decrease in the frequency of older individuals in the whole population.

The final sample involved a total of 521 healthy Italian participants (263 female, 258 male, mean age 47.7, SD 17.1, range 20–79 years old; education mean 13.21, SD 4.13, range 5–27 years). The age, education, and sex subdivisions are visible in Table 1. Informed consent forms were signed by participants before the evaluation. This study was performed in line with the principles of the Declaration of Helsinki. Approval was granted by the ethical committee of Milano-Bicocca (RM-2016-38; 10 March 2016).

### 2.2. Developmental Eye Movement (DEM) Test

DEM is a practical, easy, and inexpensive method for measuring ocular motor skills [19]. Using a simple and easy simulated reading task, the DEM test uses numbers to quantify the ability to move the eyes without an eye-tracking device. The DEM test is a paper-based oculomotor test in which the participant names numbers on three test cards with a specific layout.

The test consists of a pretest card and three test cards: two vertical tests (A and B) and one horizontal test (C).

The subjects were asked to read the numbers on the different cards as quickly as possible. For cards A and B, participants were required to read aloud the vertical columns of numbers. For card C, the participants had to read aloud the same 80 numbers horizontally in a condition analogous to text reading. The time of execution for all cards and the errors made on card C were recorded.

Four sub-scores were obtained from the DEM test. VT represents the sum of the time spent completing cards A and B. The VT is an indication of the time it takes to read aloud 80 numbers arranged vertically. AHT is the time of card C adjusted for omissions and additions. AHT measures the total time taken to read aloud 80 consecutive numbers arranged horizontally in a reading-like condition and the time spent performing saccadic eye movements between numbers. A Ratio score is calculated by dividing the AHT by the VT. The error score represents the total number of errors (omissions, additions, substitutions, and transpositions) on the C card.

### 2.3. Procedure

The experimental evaluation was conducted in a quiet and well-lit room (about 350 to 400 lux). All participants signed informed consent forms and were reviewed for inclusion/exclusion criteria, followed by the MMSE and DEM tests. The number of formal years of education refers to the number of years spent in organized, structured educational settings, such as primary and secondary schools, colleges, and universities. This is typically a measure of how long someone has been in a recognized educational system. Each participant was seated at a desk wearing the correct glasses (provided if necessary), and the different cards were positioned on a lectern at 40 cm. A stopwatch was used to quantify the number-reading time. The pretest card was positioned on the table, and the numbers were covered by a white sheet to prevent the participant from reading numbers before initiating the test. After taking the white sheet off, the examiner began recording the time when the participant began naming numbers. After the participant named the last number, the examiner stopped the stopwatch. The same procedure was repeated for the three cards using the proper instructions and reading directions. Errors were recorded on the DEM scoresheet.

### 2.4. Statistical Analyses

The analyses were divided into two sections. In the first step, descriptive and correlation analyses were conducted considering demographic variables and raw scores (Pearson, Spearman, or point-biserial correlation depending on data type). In order to assess if there are general changes with age regarding the four sub-scores of DEM, a simple age group comparison (ANOVA) was conducted for each sub-score.

In the second part, normative values were defined. Norms were defined through a regression-based procedure including several consecutive steps as performed on different neuropsychological tests [50,51,52,53] and described in detail elsewhere [54]. To find the most appropriate transformation of demographic variables (age, education, and sex) on the dependent variables (VT, AHT, Ratio, and errors, taken separately) the general linear model was used. A series of bivariate regressions were compared based on the lowest Bayesian Information Criterion (BIC) [55] and were performed to find the most effective transformations of demographic independent variables. Then, they were included in a series of bivariate and multivariate regressions with one to three predictors. Based on the lower BIC, the most appropriate regression model was then selected, only if it was significant (*p* < 0.05). BIC is a method used to choose the best model from a set of models. It considers how well the model fits the data and adds a penalty for the number of parameters to prevent overfitting. The best regression model was then applied to deviations from the mean transformed scores for independent variables (in their best transformations) and dependent variables. The correction regression equation was obtained by reversing the regression coefficients of these last regressions. Score grid corrections were developed based on the correction regression to make adjustments easy and quick for clinical use. Adjusted scores for demographic variables were obtained by adding the correction score to the raw scores.

Evaluation of normality of these scores was performed using skewness and kurtosis. If their values exceeded |1| for skewness and |3| for kurtosis, they were not considered normally distributed [56]. This approach overcomes the limitations of the Shapiro–Wilk test for a large sample size [57]. On these adjusted scores, the one-sided non-parametric 95% tolerance limits, with a confidence interval of 95%, were calculated. Percentile ranks and rank-based equivalent scores on the adjusted score were calculated [58]. All analyses were performed using the R statistical environment 4.3.2 and specific packages [59].

## 3. Results

### 3.1. Descriptive Results

**Figure 1:**
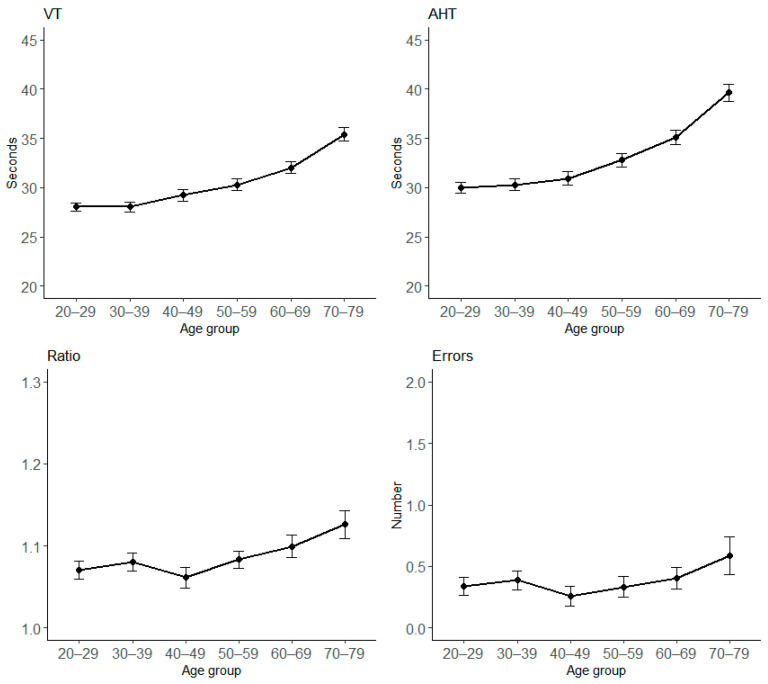
Mean scores among age groups for all DEM subtests separately. VT = Vertical Time; AHT = Adjusted Horizontal Time. Bars represent ± 1 SEM.

The descriptive raw mean results are reported in Table 2. Age differences are represented in Figure 1, which shows an increase in VT, AHT, and Ratio during the years. The age effect is medium for VT [F_(5,515)_ = 23.91, *p* < 0.0001, η^2^_p_ = 0.19] and AHT [F_(5,515)_ = 28.77, *p* < 0.0001, η^2^_p_ = 0.22], and small for Ratio [F_(5,515)_ = 3.05, *p* < 0.01, η^2^_p_ = 0.03], but not significant for errors (*p* = 0.27).

The correlation of the four DEM scores (VT, AHT, Ratio, and errors) and the three demographic indicators (age, education, and sex) are reported in Table 3. The results showed a positive moderate correlation between age and VT–AHT, and a small correlation between age and Ratio. The same pattern but negative was found for education, caused by collinearity between age and education (as reported in many studies). Errors were correlated with education and sex, and sex was not related to VT–AHT. Considering the relationship within the DEM subtest in this sample of adults compared to children [39], there is a similar high correlation between VT and AHT, but lower values when Ratio and errors were considered.

### 3.2. Normative Data Definition

The bivariate and multivariate regression selection using BIC showed that the model that better describes VT and AHT scores includes age in its cubic transformation and education in its inverse transformation. The Ratio was influenced by age in its cubic transformation. Finally, error was influenced by education in inverse transformation and sex. BIC tables used for selecting the best models are shown in Table 4.

We defined the best regression model for each subtest; to perform regression correction, the same regressions on the raw scores were redrawn from deviations from mean scores, and their coefficients were reversed. The regression to obtain correction values is reported in the caption of Table 4, Table 5, Table 6 and Table 7 for VT, AHT, Ratio, and errors, respectively. The R^2^ for each regression was 0.25, 0.29, 0.03, and 0.04 for VT, AHT, Ratio, and errors, respectively. The correction grids derived from these regression equations are available in Table 5, Table 6, Table 7 and Table 8 for quick and easy application in clinical settings. The adjusted score can be calculated by adding the raw score to the reported value obtained by the table or by regressions.

The distribution shape of adjusted scores showed non-normally distributed values for all DEM subtests (all kurtosis > 3, skewness > 1 for errors); consequently, the one-sided inner (ITL) and outer (OTL) 95% tolerance limits with 95% confidence intervals were calculated using a non-parametric approach. In a sample of 521 participants, using a score in which lower is better for all DEM subtests, the ITL and OTL correspond to the 488th and 504th observations. Their values are reported in Table 9.

The cutoff scores provided by the outer tolerance limits, together with median and other intermediate intervals, were subsequently transformed into rank-based equivalent scores (ES). Alternatively, for each score, percentiles were calculated. They are listed in Table 10 and Table 11, respectively. To facilitate scoring, the Shiny app is available at https://alessiofacchin.shinyapps.io/demadults/ (accessed on 1 February 2025).

## 4. Discussion

The aim of the present study is to determine how age-related changes affect performance on the DEM test in the adult population in addition to providing norms for this population.

Previous publications have shown the need for adult norms for the DEM test. Children with different reading disorders grow, and a single and simple test that covers all ages is useful for longitudinal monitoring and evaluation of the whole lifespan. Some studies have already administered DEM tests in neurological and neuropsychological populations, and norms based on healthy populations are required for proper patient classification [15,26,42,43].

Age-related changes in VT and AHT are consistent with expectations. The results indicated that all DEM subtest scores (VT, AHT, Ratio, and errors) were influenced by demographic variables. Specifically, VT and AHT changed significantly with age and education, showing an increase in time with age and a decrease with education. The Ratio score was influenced by age. However, the change during the years is very different, showing a large effect size for VT and AHT but medium to small for Ratio. Conversely, the number of errors decreases with higher education and they are more common in males than in females.

Previous studies showed an increasing time for vertical and horizontal subtests but a constant value over the years for the Ratio score using a modified version of DEM called Adult DEM (A-DEM) [60]. Similarly, a study based on a KD test found that scores for adults cannot be identical across the lifespan due to the influence of age and education [45]. The results of our study are in line with those of the KD test considering the small effect size found and the large sample used.

According to results obtained in developmental age, the correlation between the VT and AHT DEM subtests (internal consistency) is expected to be high. The correlation found was 0.84, perfectly in line with that previously found in children (0.85) [39]. Conversely, all other internal correlations between DEM subtests were lower than previously reported in developmental age, also confirming internal consistency in adults.

The norms defined here were reported in two standard scores: ES and percentiles. While the ES is a score prevalently used in Italy and aligned with other neuropsychological tests, reporting the percentile is important for two reasons other than its worldwide use. Firstly, it is applied in many neuropsychological tests, including those used in developmental age, including the same DEM test [48]. Secondly, it allows a more continuous and granular classification of patient performance, going beyond the simple five categories of the ES.

While the DEM can be used in adults, there are specific tests available for this population called A-DEM [60] and A-DEMd [61]. These tests use a similar paradigm but with two-digit numbers and distractors for the two tests, respectively. What makes DEM preferred over these tests? One simple reason for using DEM is related to its construction. In A-DEM and modified A-DEMd, two-digit numbers are introduced along with distractors to increase cognitive load and visual processing demands. However, in terms of content validity, to increase the measure of eye movements, it is necessary to increase the eye movement demand and reduce the naming time. As a matter of fact, both A-DEM and A-DEMd increased the cognitive demands of naming going in the opposite direction. This has motivated us to develop adult norms for the DEM test.

Having adult DEM norms offers the clinical advantage of detecting oculomotor disorders directly during neurological and neuropsychological examinations. Once difficulties are detected with this faster and easy-to-use tool, an evaluation of eye movements with eye tracking would be optimal [62]. Conversely, if no difficulties arise, it is not necessary to proceed with more sophisticated instruments.

Since DEM in development was found to be sensitive to linguistic origins, the norms developed here are specific to the Italian language. However, since the participants were adults, the different number names between languages could influence execution time less than in developmental-age participants. Since the Ratio mathematically excludes the role of naming, this score seems to be less related to linguistic origins. With these considerations, Italian norms could be useful in other languages, although specific comparisons and studies are needed to confirm this hypothesis [63].

Using the classical procedure for defining regression-based norms, it was found that the Ratio score was influenced by age and errors by education and sex. However, with high statistical power, the possibility of finding subtle influences increases, necessitating the adjustment of demographic variables when the influence is small. The small effect size found for the influence of demographic variables on Ratio and errors gives a small correction for these variables, considering them from a clinical point of view. This occurs in other cases in which the influence of demographic variables on score tests is small and a large sample is used [51]. Future psychometric studies could consider this point in depth.

In this study, a positive aspect is that DEM norms were defined based on a large sample of healthy adults using a regression-based methodology. There is a possibility that the convenience sample may represent a limitation. The majority of normative studies utilize this methodology [50,51,53,54,64] in which particular attention is paid to balancing (as much as possible) different demographic characteristics and applying specific inclusion and exclusion criteria. In future normative studies, a representative and larger sample could be used to verify and ameliorate this point. There are limitations in the lack of application of these norms to specific pathological populations, but this is exactly what research should focus on in the future. In future studies, the validity and reliability of this test in the adult population, along with its usefulness in specific neurologic disorders, need to be considered.

## 5. Conclusions

Norms for the DEM test have been provided for its application in adult populations, either pathological like those with neurological disorders, or healthy like older adults, for both clinical purposes and research.

## Figures and Tables

**Table 1:** Study sample subdivision by age, education, and sex.

Age	20–29		30–39		40–49		50–59		60–69		70–79		Total
Edu/Sex	F	M	F	M	F	M	F	M	F	M	F	M	
0–5	0	0	0	0	0	1	0	0	3	1	10	7	22
6–8	0	0	5	7	4	5	5	13	16	11	12	12	90
9–13	14	14	17	15	27	22	31	13	14	23	9	11	210
>13	36	36	23	21	17	10	12	19	4	12	4	5	199
Total	50	50	45	43	48	38	48	45	37	47	35	35	521

Note: F = females; M = males.

**Table 2:** Mean raw scores (SDs in parentheses) for each DEM subtest subdivided by age groups.

	20–29	30–39	40–49	50–59	60–69	70–79
VT	28.03 (4.05)	28.05 (4.61)	29.22 (5.32)	30.3 (5.39)	32.04 (5.64)	35.43 (5.92)
AHT	30.01 (5.25)	30.28 (5.49)	30.93 (6.26)	32.78 (6.3)	35.11 (6.44)	39.63 (7.02)
Ratio	1.07 (0.11)	1.08 (0.10)	1.06 (0.12)	1.08 (0.10)	1.1 (0.13)	1.13 (0.14)
Errors	0.34 (0.73)	0.39 (0.75)	0.26 (0.75)	0.33 (0.80)	0.4 (0.79)	0.59 (1.30)

Note: VT = Vertical Time; AHT = Adjusted Horizontal Time.

**Table 3:** Descriptive statistics (mean, SD, range or frequency) for demographic variables and the four scores of the DEM tests (VT, AHT, Ratio, and errors) together with their correlations.

	Mean (SD)	Range	Age	Education	Sex	VT	AHT	Ratio
Age	47.7 (17.1)	20–79						
Education	13.21 (4.13)	5–27	−0.43 ***					
Sex	263 F/258 M		0.02	0.07				
VT	30.28 (5.68)	17.75–51.76	0.41 ***	−0.37 ***	0.04			
AHT	32.82 (6.87)	18.34–67.20	0.44 ***	−0.41 ***	0.01	0.84 ***		
Ratio	1.09 (0.12)	0.76–1.60	0.15 ***	−0.15 ***	−0.04	−0.07	0.46 ***	
Errors	0.38 (0.86)	0–7	0.06	−0.1 *	0.17 ***	0.17 ***	0.24 ***	0.16 ***

Note: F = female; M = male; * *p* < 0.05; ** *p* < 0.01; *** *p* < 0.001. VT = Vertical Time; AHT = Adjusted Horizontal Time.

**Table 4:** Regression model comparison with the best transformation of independent variables for the four DEM scores.

Subtest	Model	K	BIC	Delta_BIC	Model Lik.	BIC Wt	LL	Cum.Wt
VT	Age + Edu	4	3164	0	1	0.92	−1569.75	0.92
	Age + Edu + Sex	5	3169	4.94	0.09	0.08	−1569.09	1
	Edu	3	3197	33.00	<0.0001	<0.0001	−1589.38	1
	Age	3	3199	34.81	<0.0001	<0.0001	−1590.28	1
	Edu + Sex	4	3201	37.25	<0.0001	<0.0001	−1588.38	1
	Age + Sex	4	3205	40.53	<0.0001	<0.0001	−1590.02	1
	Sex	3	3305	140.43	<0.0001	<0.0001	−1643.09	1
AHT	Age + Edu	4	3335	0	1	0.96	−1655.06	0.96
	Age + Edu + Sex	5	3341	6.19	0.05	0.04	−1655.03	1
	Age	3	3371	36.01	<0.0001	<0.0001	−1676.2	1
	Age + Sex	4	337	42.25	<0.0001	<0.0001	−1676.19	1
	Edu	3	3384	48.97	<0.0001	<0.0001	−1682.68	1
	Edu + Sex	4	3390	54.86	<0.0001	<0.0001	−1682.49	1
	Sex	3	3504	168.85	<0.0001	<0.0001	−1742.62	1
Ratio	Age	3	−749	0	1	0.72	384.3399	0.72
	Age + Edu	4	−746	3.13	0.21	0.15	385.9038	0.86
	Edu	3	−745	4.84	0.09	0.06	381.9176	0.93
	Age + Sex	4	−744	5.05	0.08	0.06	384.9443	0.99
	Age + Edu + Sex	5	−741	8.46	0.01	0.01	386.3632	0.99
	Edu + Sex	4	−739	10.49	0.01	<0.01	382.2221	1
	Sex	3	−734	15.90467	<0.001	<0.001	376.3876	1
Errors	Edu + Sex	4	1318	0	1	0.63	−646.632	0.63
	Sex	3	1319	1.58	0.45	0.29	−650.549	0.92
	Age + Sex	4	1323	5.08	0.08	0.05	−649.173	0.97
	Age + Edu + Sex	5	1324	6.18	0.05	0.03	−646.596	1
	Edu	3	1329	10.95	<0.005	<0.005	−655.235	1
	Age	3	1333	14.71	<0.001	<0.0005	−657.115	1
	Age + Edu	4	1335	16.98	<0.001	<0.0005	−655.123	1

Note: K = number of parameters of the model; BIC = Bayesian Information Criterion; Delta BIC = BIC difference between the best model and the model listed; Model Lik. = the relative likelihood of the model; BIC Wt = model probabilities; LL = log-likelihood of the model; Cum. Wt = cumulative weights. The best model selected is shown in the first line for each DEM subtest. VT = Vertical Time; AHT = Adjusted Horizontal Time.

**Table 5:** Correction grid for computing the adjusted scores of VT.

Edu/Age	20	25	30	35	40	45	50	55	60	65	70	75
8	−0.2	−0.29	−0.42	−0.61	−0.87	−1.19	−1.6	−2.1	−2.69	−3.39	−4.22	−5.16
13	2.12	2.02	1.89	1.7	1.44	1.12	0.71	0.22	−0.38	−1.08	−1.9	−2.85
16	2.81	2.72	2.58	2.39	2.14	1.81	1.41	0.91	0.31	−0.39	−1.21	−2.16
18	3.14	3.05	2.92	2.72	2.47	2.15	1.74	1.24	0.65	−0.06	−0.88	−1.82
21	3.52	3.43	3.3	3.11	2.85	2.53	2.12	1.62	1.03	0.33	−0.5	−1.44

Note: The adjusted score can be calculated by adding the raw score to the reported value obtained by the table. Age and education should be selected based on the nearest values. If precise scoring is required, the correction regression should be used. Adjusted score = raw score − 0.000012 × ((Age^3^ − 150,382.9) − 48.074) × (1/Education − 0.0854).

**Table 6:** Correction grid for computing the adjusted scores of AHT.

Edu./Age	20	25	30	35	40	45	50	55	60	65	70	75
8	0.14	0.01	−0.18	−0.45	−0.81	−1.27	−1.85	−2.55	−3.39	−4.39	−5.55	−6.89
13	2.91	2.78	2.58	2.31	1.96	1.49	0.92	0.22	−0.63	−1.62	−2.79	−4.13
16	3.74	3.61	3.41	3.14	2.79	2.32	1.75	1.04	0.2	−0.8	−1.96	−3.3
18	4.14	4.01	3.81	3.54	3.18	2.72	2.15	1.44	0.6	−0.4	−1.56	−2.9
21	4.59	4.46	4.27	4	3.64	3.18	2.6	1.9	1.06	0.06	−1.1	−2.44

Note: The adjusted score can be calculated by adding the raw score to the reported value obtained by the table. Age and education should be selected based on the nearest values. If precise scoring is required, the correction regression should be used. Adjusted score = raw score − 0.000017 × ((Age^3^ − 150,382.9) − 57.49) × (1/Education − 0.0854).

**Table 7:** Correction grid for computing the adjusted scores of Ratio.

Age	F20	F25	F30	F35	F40	F45	F50	F55	F60	F65	F70	F75
	0.02	0.02	0.02	0.02	0.01	0.01	0	0	−0.01	−0.02	−0.03	−0.04

Note: The adjusted score can be calculated by adding the raw score to the reported value obtained by the table. Age and education should be selected based on the nearest values. If precise scoring is required, the correction regression should be used. Adjusted score = raw score − 0.00000016 × ((Age^3^) − 150,382.9).

**Table 8:** Correction grid for computing the adjusted scores of errors.

Education	M	F
8	−0.3	0
13	−0.1	0.2
16	−0.1	0.2
18	−0.1	0.2
21	0	0.3

Note: The adjusted score can be calculated by adding the raw score to the reported value obtained by the table. Age and education should be selected based on the nearest values. If precise scoring is required, the correction regression should be used. Adjusted score = raw score − 3.02 × (1/Education) − 0.0854) − 0.307 × (Sex − 0.495). Sex was coded as male = 1 and female = 0.

**Table 9:** Internal tolerance limits (ITLs) and outer tolerance limits (OTLs) for the DEM subtest.

Subtest	ITL	OTL
VT	38.1	40.4
AHT	41.8	45.3
Ratio	1.33	1.26
Errors	2.2	1.9

**Table 10:** Equivalent scores (ES) for the four subtests of the DEM test.

Subtest	PE = 0	PE = 1	PE = 2	PE = 3	PE = 4
VT	≥40.4	40.3–34.2	34.1–31.9	31.8–29.9	<29.9
AHT	≥45.3	45.2–37.7	37.6–34.7	34.6–32.0	<32.0
Ratio	≥1.33	1.32–1.18	1.17–1.12	1.11–1.08	<1.08
Errors	≥2.2	2.1–0.9	0.8–0.2	0.8–0.2	<0.2

Note: Data are based on the adjusted score. VT = Vertical Time; AHT = Adjusted Horizontal Time.

**Table 11:** Percentile scoring table for the four subtests of the DEM test.

Percentile	VT	AHT	Ratio	Errors
99	20.9	22.1	0.83	−0.5
95	22.8	24.7	0.93	−0.3
90	24.2	26.2	0.95	−0.2
85	25.3	27.1	0.98	−0.1
80	26.2	27.9	1	−0.1
75	26.8	28.6	1.02	−0.1
70	27.5	29.3	1.02	−0.1
65	28.2	30.1	1.03	0
60	28.9	30.8	1.05	0
55	29.4	31.5	1.06	0.1
50	29.9	32	1.08	0.2
45	30.5	33	1.09	0.2
40	31	33.9	1.1	0.2
35	31.7	34.7	1.12	0.2
30	32.5	35.4	1.13	0.2
25	33.2	36.3	1.15	0.3
20	33.9	37.5	1.16	0.9
15	35.2	38.5	1.19	0.9
10	36.7	39.8	1.23	1.2
5	39.2	43.4	1.29	1.9
4	40	44.2	1.31	2.1
3	40.5	45.5	1.34	2.2
2	41.2	48	1.37	2.9
1	43.3	49.1	1.41	3.9

Note: Data are based on the adjusted score. VT = Vertical Time; AHT = Adjusted Horizontal Time.

## Data Availability

The data presented in this study are available on request from the corresponding author. The data are not publicly available due to restrictions included in the informed consent provided by participants.

## References

[1] Jan J.E., Lyons C.J., Heaven R.K., Matsuba C. (2001). Visual Impairment Due to a Dyskinetic Eye Movement Disorder in Children with Dyskinetic Cerebral Palsy. Dev. Med. Child Neurol..

[2] Anderson T.J., MacAskill M.R. (2013). Eye Movements in Patients with Neurodegenerative Disorders. Nat. Rev. Neurol..

[3] Serra A., Chisari C.G., Matta M. (2018). Eye Movement Abnormalities in Multiple Sclerosis: Pathogenesis, Modeling, and Treatment. Front. Neurol..

[4] Poletti B., Solca F., Carelli L., Diena A., Colombo E., Torre S., Maranzano A., Greco L., Cozza F., Lizio A. (2021). Association of Clinically Evident Eye Movement Abnormalities With Motor and Cognitive Features in Patients With Motor Neuron Disorders. Neurology.

[5] Facchin A., Buonocore J., Crasà M., Quattrone A., Quattrone A. (2024). Systematic Assessment of Square-Wave Jerks in Progressive Supranuclear Palsy: A Video-Oculographic Study. J. Neurol..

[6] Thiagarajan P., Ciuffreda K.J., Capo-Aponte J.E., Ludlam D.P., Kapoor N. (2014). Oculomotor Neurorehabilitation for Reading in Mild Traumatic Brain Injury (mTBI): An Integrative Approach. NeuroRehabilitation.

[7] Manto M., Triarhou L.C. (2023). Ocular Dysmetria, Flutter, and Opsoclonus: Refining the Oculomotor Deficits in Cerebellar Patients. Cerebellum.

[8] Rodríguez-Labrada R., Vázquez-Mojena Y., Velázquez-Pérez L., Rodríguez-Labrada R., Vázquez-Mojena Y., Velázquez-Pérez L. (2019). Eye Movement Abnormalities in Neurodegenerative Diseases. Eye Motility.

[9] Pretegiani E., Optican L.M. (2017). Eye Movements in Parkinson’s Disease and Inherited Parkinsonian Syndromes. Front. Neurol..

[10] Gorges M., Pinkhardt E.H., Kassubek J. (2014). Alterations of Eye Movement Control in Neurodegenerative Movement Disorders. J. Ophthalmol..

[11] Farashi S. (2021). Analysis of Vertical Eye Movements in Parkinson’s Disease and Its Potential for Diagnosis. Appl. Intell..

[12] Quattrone A., Crasà M., Morelli M., Vescio B., Augimeri A., Gramigna V., Quattrone A. (2022). Video-Oculographic Biomarkers for Evaluating Vertical Ocular Dysfunction in Progressive Supranuclear Palsy. Park. Relat. Disord..

[13] Liu Z., Yang Z., Gu Y., Liu H., Wang P. (2021). The Effectiveness of Eye Tracking in the Diagnosis of Cognitive Disorders: A Systematic Review and Meta-Analysis. PLoS ONE.

[14] Höglinger G.U., Respondek G., Stamelou M., Kurz C., Josephs K.A., Lang A.E., Mollenhauer B., Müller U., Nilsson C., Whitwell J.L. (2017). Clinical Diagnosis of Progressive Supranuclear Palsy: The Movement Disorder Society Criteria. Mov. Disord..

[15] Facchin A. (2021). Spotlight on the Developmental Eye Movement (DEM) Test. Clin. Optom..

[16] Galetta K.M., Liu M., Leong D.F., Ventura R.E., Galetta S.L., Balcer L.J. (2016). The King-Devick Test of Rapid Number Naming for Concussion Detection: Meta-Analysis and Systematic Review of the Literature. Concussion.

[17] Galetta K.M., Barrett J., Allen M., Madda F., Delicata D., Tennant A.T., Branas C.C., Maguire M.G., Messner L.V., Devick S. (2011). The King-Devick Test as a Determinant of Head Trauma and Concussion in Boxers and MMA Fighters. Neurology.

[18] Silverberg N.D., Luoto T.M., Öhman J., Iverson G.L. (2014). Assessment of Mild Traumatic Brain Injury with the King-Devick Test^®^ in an Emergency Department Sample. Brain Inj..

[19] Garzia R.P., Richman J.E., Nicholson S.B., Gaines C.S. (1990). A New Visual-Verbal Saccade Test: The Developmental Eye Movement Test (DEM). J. Am. Optom. Assoc..

[20] Richman J.E. (2008). The Developmental Eye Movement Test (DEM) 2.0 Manual.

[21] Tassinari J.T., DeLand P. (2005). Developmental Eye Movement Test: Reliability and Symptomatology. Optometry.

[22] Gil-Casas A., Piñero-Llorens D.P., Molina-Martín A. (2022). Developmental Eye Movement (DEM) and King-Devick (K-D) Performance in Multiple Sclerosis. Brain Sci..

[23] López-de-la-Fuente C., Saz-Onrubia E., Orduna-Hospital E., Sánchez-Cano A. (2024). Comparison of Two Visual-Verbal Tests of Ocular Motility Using an Eye-Tracker. J. Optom..

[24] Heick J.D., Bay C., Valovich McLeod T.C. (2018). Evaluation of Vertical and Horizontal Saccades Using the Developmental Eye Movement Test Compared to the King-Devick Test. Int. J. Sports Phys. Ther..

[25] Ibrahimi D., Aviles M., Rodríguez-Reséndiz J. (2024). Oculomotor Patterns in Children with Poor Reading Abilities Measured Using the Development Eye Movement Test. J. Clin. Med..

[26] Powell J.M., Birk K., Cummings E.H., Ciol M.A. (2005). The Need for Adult Norms on the Developmental Eye Movement Test. J. Behav. Optom..

[27] Han Y., Ciuffreda K.J., Kapoor N. (2004). Reading-Related Oculomotor Testing and Training Protocols for Acquired Brain Injury in Humans. Brain Res. Protoc..

[28] Kapoor N., Ciuffreda K.J. (2018). Assessment of Neuro-Optometric Rehabilitation Using the Developmental Eye Movement (DEM) Test in Adults with Acquired Brain Injury. J. Optom..

[29] Guantay C.D., Mena-García L., Tola-Arribas M.Á., Garea García-Malvar M.J., Para-Prieto M., González Fernández G., Mayo-Iscar A., Pastor J.C. (2024). Accounting for Visual Field Abnormalities When Using Eye-Tracking to Diagnose Reading Problems in Neurological Degeneration. J. Eye Mov. Res..

[30] Ciuffreda K.J., Rutner D., Kapoor N., Suchoff I.B., Craig S., Han M.E. (2008). Vision Therapy for Oculomotor Dysfunctions in Acquired Brain Injury: A Retrospective Analysis. Optom.—J. Am. Optom. Assoc..

[31] Ayton L.N., Abel L.A., Fricke T.R., McBrien N.A. (2009). Developmental Eye Movement Test: What Is It Really Measuring?. Optom. Vis. Sci..

[32] Webber A., Wood J., Gole G., Brown B. (2011). DEM Test, Visagraph Eye Movement Recordings, and Reading Ability in Children. Optom. Vis. Sci..

[33] Tanke N., Barsingerhorn A.D., Boonstra F.N., Goossens J. (2021). Visual Fixations Rather than Saccades Dominate the Developmental Eye Movement Test. Sci. Rep..

[34] Wood J.M., Black A.A., Hopkins S., White S.L.J. (2018). Vision and Academic Performance in Primary School Children. Ophthalmic Physiol. Opt..

[35] Hopkins S., Black A.A., White S.L.J., Wood J.M. (2019). Visual Information Processing Skills Are Associated with Academic Performance in Grade 2 School Children. Acta Ophthalmol..

[36] Hindmarsh G.P., Black A.A., White S.L., Hopkins S., Wood J.M. (2021). Eye Movement Patterns and Reading Ability in Children. Ophthalmic Physiol. Opt..

[37] Northway N. (2003). Predicting the Continued Use of Overlays in School Children—A Comparison of the Developmental Eye Movement Test and the Rate of Reading Test. Ophthalmic Physiol. Opt..

[38] Palomo-Álvarez C., Puell M.C. (2009). Relationship between Oculomotor Scanning Determined by the DEM Test and a Contextual Reading Test in Schoolchildren with Reading Difficulties. Graefes Arch. Clin. Exp. Ophthalmol..

[39] Facchin A., Maffioletti S., Carnevali T. (2011). Validity Reassessment of Developmental Eye Movement (DEM) Test in the Italian Population. Optom. Vis. Dev..

[40] Serdjukova J., Ekimane L., Valeinis J., Skilters J., Krumina G. (2017). How Strong and Weak Readers Perform on the Developmental Eye Movement Test (DEM): Norms for Latvian School-Aged Children. Read. Writ..

[41] Facchin A., Maffioletti S. (2018). The Reliability of the DEM Test in the Clinical Environment. Front. Psychol..

[42] Radomski M.V., Finkelstein M., Llanos I., Scheiman M., Wagener S.G. (2014). Composition of a Vision Screen for Servicemembers With Traumatic Brain Injury: Consensus Using a Modified Nominal Group Technique. Am. J. Occup. Ther..

[43] Suchoff I.B., Kapoor N., Waxman R., Ference W. (1999). The Occurrence of Ocular and Visual Dysfunctions in an Acquired Brain-Injured Patient Sample. J. Am. Optom. Assoc..

[44] Powell J.M., Fan M.-Y., Kiltz P.J., Bergman A.T., Richman J. (2006). A Comparison of the Developmental Eye Movement Test (DEM) and a Modified Version of the Adult Developmental Eye Movement Test (A-DEM) with Older Adults. J. Behav. Optom..

[45] Anderson H.D., Biely S.A. (2017). Baseline King–Devick Scores for Adults Are Not Generalizable; However, Age and Education Influence Scores. Brain Inj..

[46] Chen A.-H., Khalid N.M., Buari N.H. (2019). Age Factor Affects Reading Acuity and Reading Speed in Attaining Text Information. Int. J. Ophthalmol..

[47] Pegoraro S., Facchin A., Luchesa F., Rolandi E., Guaita A., Arduino L.S., Daini R. (2024). The Complexity of Reading Revealed by a Study with Healthy Older Adults. Brain Sci..

[48] Facchin A., Maffioletti S., Carnevali T. (2012). The Developmental Eye Movement (DEM) Test: Normative Data for Italian Population. Optom. Vis. Dev..

[49] Measso G., Cavarzeran F., Zappalà G., Lebowitz B.D., Crook T.H., Pirozzolo F.J., Amaducci L.A., Massari D., Grigoletto F. (1993). The Mini-mental State Examination: Normative Study of an Italian Random Sample. Dev. Neuropsychol..

[50] Rigoli M., Facchin A., Cardile D., Beschin N., Luzzatti C. (2021). Open-Source Open-Access Reaction Time Test (OORTT): An Easy Tool to Assess Reaction Times. Neurol. Sci..

[51] Facchin A., Simioni M., Maffioletti S., Daini R. (2023). Broken Ring enVision Search (BReViS): A New Clinical Test of Attention to Assess the Effect of Layout and Crowding on Visual Search. Brain Sci..

[52] Capitani E., Laiacona M. (2017). Outer and Inner Tolerance Limits: Their Usefulness for the Construction of Norms and the Standardization of Neuropsychological Tests. Clin. Neuropsychol..

[53] Aiello E.N., Gramegna C., Esposito A., Gazzaniga V., Zago S., Difonzo T., Maddaluno O., Appollonio I., Bolognini N. (2022). The Montreal Cognitive Assessment (MoCA): Updated Norms and Psychometric Insights into Adaptive Testing from Healthy Individuals in Northern Italy. Aging Clin. Exp. Res..

[54] Facchin A., Mischi E., Iannello C., Maffioletti S., Daini R. (2022). Normative Values of the Groffman Visual Tracing Test for the Assessment of Oculomotor Performance in the Adult Population. Vision.

[55] Zhang J., Yang Y., Ding J. (2023). Information Criteria for Model Selection. WIREs Comput. Stat..

[56] Kim H.-Y. (2013). Statistical Notes for Clinical Researchers: Assessing Normal Distribution (2) Using Skewness and Kurtosis. Restor Dent. Endod..

[57] Mishra P., Pandey C.M., Singh U., Gupta A., Sahu C., Keshri A. (2019). Descriptive Statistics and Normality Tests for Statistical Data. Ann. Card. Anaesth..

[58] Facchin A., Rizzi E., Vezzoli M. (2022). A Rank Subdivision of Equivalent Score for Enhancing Neuropsychological Test Norms. Neurol. Sci..

[59] R Core Team (2024). R: A Language and Environment for Statistical Computing.

[60] Sampedro A., Richman J.E., Pardo M.S. (2003). The Adult Developmental Eye Movement Test (ADEM) a Tool for Saccadic Evaluation in Adults. J. Behav. Optom..

[61] Gené-Sampedro A., Monteiro P.M.L., Bueno-Gimeno I., Gene-Morales J., Piñero D.P. (2021). Validation of a Modified Version of the Adult Developmental Eye Movement Test. Sci. Rep..

[62] Ceple I., Krauze L., Serpa E., Svede A., Goliskina V., Vasiljeva S., Kassaliete E., Ganebnaya A., Volberga L., Truksa R. (2025). Eye Movement Parameters in Children with Reading Difficulties. Appl. Sci..

[63] Ben-Eli H., Blique H., Scheiman M., Eichler R. (2025). Developmental Eye Movement Test Results of Hebrew-Speaking Children with Cross-Linguistic Comparisons. Ophthalmic Physiol. Opt..

[64] Facchin A., Vallar G., Daini R. (2021). The Brentano Illusion Test (BRIT): An Implicit Task of Perceptual Processing for the Assessment of Visual Field Defects in Neglect Patients. Neuropsychol. Rehabil..

